# Age-related striatal BOLD changes without changes in behavioral loss aversion

**DOI:** 10.3389/fnhum.2015.00176

**Published:** 2015-04-30

**Authors:** Vijay Viswanathan, Sang Lee, Jodi M. Gilman, Byoung Woo Kim, Nick Lee, Laura Chamberlain, Sherri L. Livengood, Kalyan Raman, Myung Joo Lee, Jake Kuster, Daniel B. Stern, Bobby Calder, Frank J. Mulhern, Anne J. Blood, Hans C. Breiter

**Affiliations:** ^1^Medill Integrated Marketing Communications, Northwestern UniversityEvanston, IL, USA; ^2^Applied Neuromarketing Consortium: Northwestern University, Wayne State University, University of Michigan, Loughborough University School of Business and Economics (UK) and Massachusetts General Hospital/Harvard UniversityChicago, IL, USA; ^3^Mood and Motor Control Laboratory or Laboratory of Neuroimaging and Genetics, Department of Psychiatry, Massachusetts General HospitalBoston, MA, USA; ^4^Warren Wright Adolescent Center, Department of Psychiatry and Behavioral Science, Northwestern University Feinberg School of MedicineChicago, IL, USA; ^5^Northwestern University and Massachusetts General Hospital Phenotype Genotype Project in Addiction and Mood DisordersChicago, IL, USA; ^6^Marketing Group, Aston Business SchoolBirmingham, UK; ^7^Department of Marketing, Kellogg School of Management, Northwestern UniversityEvanston, IL, USA

**Keywords:** loss aversion, aging, nucleus accumbens, reward, fMRI, neurocompensation

## Abstract

Loss aversion (LA), the idea that negative valuations have a higher psychological impact than positive ones, is considered an important variable in consumer research. The literature on aging and behavior suggests older individuals may show more LA, although it is not clear if this is an effect of aging in general (as in the continuum from age 20 and 50 years), or of the state of older age (e.g., past age 65 years). We also have not yet identified the potential biological effects of aging on the neural processing of LA. In the current study we used a cohort of subjects with a 30 year range of ages, and performed whole brain functional MRI (fMRI) to examine the ventral striatum/nucleus accumbens (VS/NAc) response during a passive viewing of affective faces with model-based fMRI analysis incorporating behavioral data from a validated approach/avoidance task with the same stimuli. Our* a priori* focus on the VS/NAc was based on (1) the VS/NAc being a central region for reward/aversion processing; (2) its activation to both positive and negative stimuli; (3) its reported involvement with tracking LA. LA from approach/avoidance to affective faces showed excellent fidelity to published measures of LA. Imaging results were then compared to the behavioral measure of LA using the same affective faces. Although there was no relationship between age and LA, we observed increasing neural differential sensitivity (NDS) of the VS/NAc to avoidance responses (negative valuations) relative to approach responses (positive valuations) with increasing age. These findings suggest that a central region for reward/aversion processing changes with age, and may require more activation to produce the same LA behavior as in younger individuals, consistent with the idea of neural efficiency observed with high IQ individuals showing less brain activation to complete the same task.

## Introduction

Age is among the most commonly used variables in marketing and consumer research. While age is a deceptively simple variable, the underlying construct of biological age and how it relates to behavior is not always clear. One age effect supported by a number of social psychology studies is that older adults put more weight on avoiding potential negative outcomes, as evidenced by an aversion to change (Botwinick, 1978) and nostalgia for early experience (Schindler and Holbrook, 2003). Aging research points to an association of age with making less risky decisions (Johnson and Busemeyer, 2010), and suggests that older individuals generally avoid losses to a greater extent than younger individuals (e.g., Heckhausen, 1997). A fundamental way to quantify this perspective is with the concept of “loss aversion” (LA), in which negative stimuli have a disproportionate psychological impact relative to positive ones (Kahneman and Tversky, 1979), and can be defined mathematically by the ratio of valuation of monetary losses relative to valuation of gains (Tversky and Kahneman, 1991), or in more general terms, as the ratio of avoidance to approach measures (Abdellaoui et al., 2007). LA has become an important variable in consumer research (Ariely et al., 2005; Paraschiv and L’Haridon, 2008) and is consistent with the observation that older individuals are more focused on goals pertaining to maintenance and regulation of loss (Ebner et al., 2006). Cole et al. (2008) suggest, based on “regulatory focus theory” (Avnet and Higgins, 2006), that older individuals would be more prevention-focused i.e., avoid losses, than promotion-focused i.e., pursuit of gains.

Neuroscience studies have examined the biological basis for age-related changes in cognitive function (Hedden and Gabrieli, 2004; Mohr et al., 2010), which might affect biases in decision-making such as LA. For instance, Raz (2000) found a steady decline in the prefrontal cortex (PFC) structures starting from the age of 20 along with a decline in the striatal volume over the lifespan of an individual. In many studies, the biology of age-related changes in the brain goes in the same direction as behavior (Good et al., 2001), as for instance in the domain of episodic memory, where older adults have demonstrated decreased activation of various sites in the left and right prefrontal cortices correlating with decreased performance on the task relative to their younger counterparts (Grady et al., 1995, 1999; Cabeza et al., 1997; Madden et al., 1999; Grady and Craik, 2000; Reuter-Lorenz, 2002; Stebbins et al., 2002). An alternate outcome is also possible, wherein alterations in brain activity are not associated with an alteration of behavior, namely, increasing amounts of activation are needed to produce the same behavior (e.g., neurocompensation; Cabeza et al., 2002; Park and Reuter-Lorenz, 2009; Daselaar et al., 2015).

These neuroimaging and behavioral studies thus suggest at least two potential hypotheses regarding LA, its underlying neural substrate, and aging: (1) LA behavior may parallel changes in neural processing, specifically, LA behavior may increase with age along with increased activation in tissue required to process it; or (2) LA behavior may increase more slowly than the compensatory activity in tissue processing it (i.e., there may be small differences in LA behavior with age, and strong brain activity differences during its processing). This latter possibility finds support from an early functional MRI (fMRI) study that reported decreases in either performance or IQ were associated with increased brain activation during cognitive function (Seidman et al., 1998), and the observation of potentially compensatory activity in older individuals (Meunier et al., 2014). Two recent studies of LA specifically support option (2) above, in that they report LA behavior does not change between young adults and old adults (Li et al., 2013) or between adolescents and young adults (Barkley-Levenson et al., 2013). One of these studies also evaluated neural processing of LA, and found differences between adolescents and young adults in large decision-making networks, further supporting option (2) (Barkley-Levenson et al., 2013).

In the current study we sought to test these hypotheses evaluating subject age against (1) the relative overweighting of behavioral responses to negative vs. positive stimuli (i.e., LA behavior) using a validated keypress measure (Kim et al., 2010); and (2) neural differential sensitivity (NDS; Tom et al., 2007) within the ventral striatum/nucleus accumbens (VS/NAc) to the same stimuli used in the behavior task. Given the substantial involvement of the VS/NAc in motor preparation (e.g., Florio et al., 1999) and abnormality with motor illnesses such as Parkinsonism (e.g., Aarts et al., 2014; Payer et al., 2015), we sought to avoid the motoric contamination inherent with cognitive imaging studies of the VS/NAc using monetary choice paradigms (Tom et al., 2007; Canessa et al., 2013). Since the motor responses for the keypress task would not be separate from reward/aversion assessments (the amount of VS/NAc activation could just reflect how much an individual was keypressing to approach or avoid a stimulus, or reflect the urgency of their responses), the keypress task was done outside the MRI, and the outcome of keypress responses used for model-based analysis of VS/NAc signal during passive viewing of the same stimuli; such a model-based approach to fMRI has been used with this task (Aharon et al., 2001) and fMRI-based imaging-genetics used with these stimuli and task before (Perlis et al., 2008; Gasic et al., 2009), consistent with the framework for model-based imaging discussed by others (e.g., Mittner et al., 2014; Wang and Voss, 2014; White et al., 2014; Xu et al., 2015). The implicit assumption in this model-based application was that the emotional response to faces in the scanner, and the behavior based on emotional response to the same stimuli outside of the scanner would be related, as suggested for studies of emotion-based processing by other investigators (Hayes and Northoff, 2012).

To examine the underlying physiological effects of age on LA, we performed whole brain fMRI to monitor activity within the VS/NAc, using a passive viewing paradigm with affective faces known to evoke positive and negative valuations (Strauss et al., 2005), given the VS/NAc is a central region for reward/aversion processing (Breiter et al., 1997; Blood et al., 1999; Breiter and Rosen, 1999; Hayes and Northoff, 2012), and has been shown to activate to both positive and negative stimuli (Aharon et al., 2001; Becerra et al., 2001; Breiter et al., 2001; Kober et al., 2008; Hayes and Northoff, 2012) and to track LA for the choice and anticipation phases of decision making (Tom et al., 2007; Lee et al., 2012; Canessa et al., 2013). For a behavioral index of LA, we used the same affective faces (Ekman and Friesen, 1976) with a keypress task performed outside the MRI that allowed the subject multiple potential decisions: (1) to do nothing about the default viewing time of a picture; (2) to view the picture for longer (approach); or (3) to view the picture for shorter (avoidance) time. The keypress data was analyzed to produce a value function (Breiter and Kim, 2008; Kim et al., 2010) for each subject that is analogous to a prospect theory value function or utility curve (Kahneman and Tversky, 1979), but unlike any other reward/aversion construct actually uses an entropy variable representing information (Shannon and Weaver, 1949). The slopes of the negative and positive portions of this curve can be readily sampled to yield a measure of LA in a general framework for LA as an overweighting of aversion (toward negative stimuli) relative to approach (toward positive stimuli) as discussed by Abdellaoui et al. (2007). For the model-based fMRI analysis, we explicitly required that (i) relative activation to negative (avoidance) stimuli vs. positive (approach) stimuli (i.e., the NDS of avoidance vs. approach) would occur in the VS/NAc; and (ii) that NDS-related fMRI signal in the VS/NAc would significantly correlate with LA behavior across subjects. If this were observed, we then sought to evaluate if VS/NAc NDS would increase with age, and whether or not it would parallel any relationship of LA behavior to age, supporting either the first or second hypothesis regarding the interaction of LA, its underlying neural substrate, and aging.

## Methods

### Subjects

Healthy control subjects were aggregated for an exploratory analysis on an available sample of subjects with complete behavior and imaging data from three paradigms for which LA parameters could be computed and evaluated. The resulting sample of 17 subjects was compiled for use with another project testing if the negative component of LA could explain aspects of amygdala function across the three paradigms and connect it to structural measures (Lee et al., 2012); the current study focused just on the emotional faces paradigm given the value function curves from keypressing to these stimuli had never been published, nor evaluated against NDS or age. These 17 subjects were recruited by advertisement and were part of a larger phenotype genotype project in addiction and mood disorder (PGP)^1^. Subjects were free of any psychiatric, neurological, or medical issues per psychiatrist-based SCID for DSM-IV diagnoses, medical review of systems and physical evaluation including blood chemistry. Race was determined by individual self-identification using a standardized form (Benson and Marano, 1998), and handedness via the Edinburgh Handedness Inventory (Oldfield, 1971). Participating subjects were without any current or lifetime DSM-IV Axis I disorder or major medical illness known to influence brain structure or function, including neurologic disease, HIV, and hepatitis C. Subjects were scanned at normal or corrected normal vision. Women were scanned during their mid-follicular phase based upon self-reported menstrual history, with confirmation at the time of scanning based on hormonal testing with a urine assay.

Participants in the study were adults (10 males, 7 females; 5 African Americans and 12 Caucasians) between the ages of 20 and 55 with a mean (±SE) age of 35.8 ± 2.7 years, with no significant difference between men and women (*F*_(1,15)_ = 1.81, *P* < 0.20). They had a mean educational history of 15.4 ± 1.9 years, with no significant difference between men and women (*F*_(1,15)_ = 2.78, *P* < 0.12). Fifteen of subjects were right-handed.

### Experimental Paradigm and Offline Behavioral Testing

#### In Scanner

**Figure 1:**
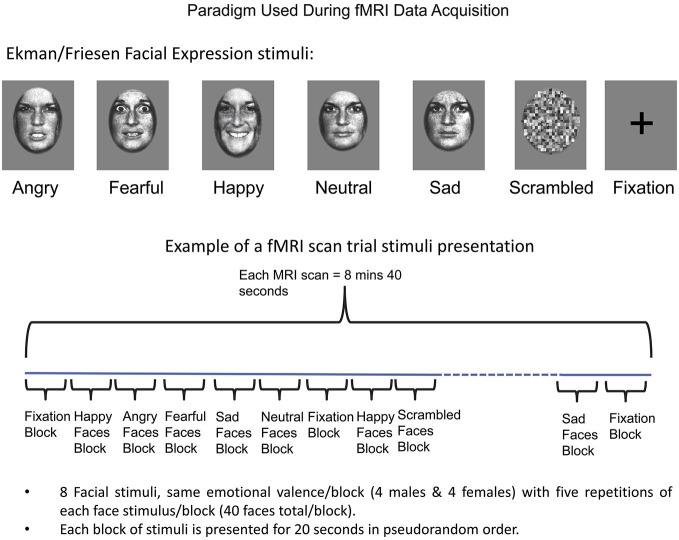
**Schema for Experimental Paradigm Used with fMRI**. At top, the categories of facial expressions used are shown along with baselines (scrambled faces and fixation). A schema of how these stimuli were presented as blocks is shown at bottom, along with details relating to number of stimuli used per block. See Methods Section for further detail.

Two fMRI scans were acquired (8 min 40 s each), each consisting of 20-s blocks of the following seven experimental conditions: angry, fearful, happy, sad, neutral expressions (Ekman and Friesen, 1976), along with phase-scrambled stimuli and fixation (Figure 1). During each scan, the seven conditions (blocks) were presented in a counterbalanced order such that no condition followed or preceded another more than once. This produced a sequence of 25 blocks for the first run, and 24 plus one blocks for the second run, with the extra block in the second run being equivalent to the last block in the first run, placed at the beginning to maintain counterbalancing across all conditions. Each facial expression block included standardized images of faces of eight individuals (four males) in a pseudorandom order (Breiter et al., 1996; Strauss et al., 2005). Each face was displayed for 200 ms with a 300 ms interstimulus interval during which a fixation cross was displayed, with five repetitions of each face stimulus per block (40 faces total per block). Face stimuli (Ekman and Friesen, 1976) were previously normalized at the MIT Media Lab (Breiter et al., 1996). The face stimuli were projected via a Sharp XG-2000V color LCD projector through a collimating lens onto a hemicircular tangent screen and viewed by the subject via a mirror affixed to the head coil. Subjects were instructed to simply look at the faces, keeping their eyes focused on the center of the picture at the location of the cross-hair. After completion of scanning, subjects performed a memory task in which they were asked to identify faces and facial expressions they had seen during the scanning session.

#### Offline Behavioral Testing

**Figure 2:**
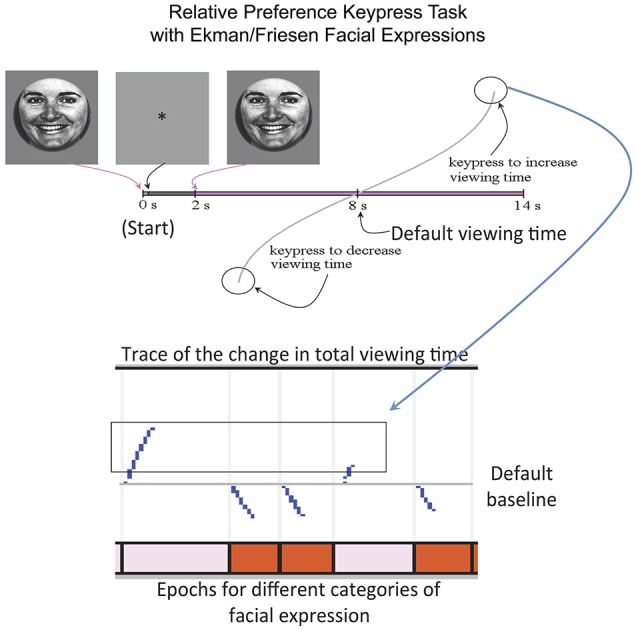
**Keypress Paradigm**. The behavioral task done outside the MRI to minimize motor confounds to activation is schematized above, with an example raster plot below.

This experiment utilized a keypress task to determine each subject’s relative preference toward the ensemble of faces (Aharon et al., 2001; Elman et al., 2005; Strauss et al., 2005; Levy et al., 2008; Makris et al., 2008; Perlis et al., 2008; Gasic et al., 2009; Yamamoto et al., 2009; Kim et al., 2010), which had been used for passive viewing during scanning. The separation of passive viewing and keypress response allowed the fMRI component to be free of motoric elements, which would otherwise confound interpretation of the fMRI results (please see Introduction Section). The keypress procedure was implemented with MatLab software on a PC (i.e., a personal computer). This task captured the reward valuation attributed to each observed face, and quantified positive and negative preferences involving (i) decision-making regarding the valence of behavior; and (ii) judgments that determine the magnitude of approach and avoidance (Breiter et al., 2006; Perlis et al., 2008; Figure 2). The objective was to determine how much effort each subject was willing to trade for viewing each facial expression compared to a default viewing time. Subjects were told that they would be exposed to a series of pictures that would change every 8 s (the default valuation of 6 s + 2 s decision block; Figure 2) if they pressed no keys. If they wanted a picture to disappear faster, they could alternate pressing one set of keys (#3 and #4 on the button box), whereas if they wanted a picture to stay longer on the screen, they could alternate pressing another set of keys (#1 and #2 on the button box). Subjects had a choice to do nothing (default condition), increase viewing time, decrease viewing time, or a combination of the two responses (Figure 2). A “slider” was displayed to the left of each picture to indicate total viewing time. Subjects were informed that the task would last approximately 20 min, and that this length was independent of their behavior, as was their overall payment. The dependent measure of interest was the amount of work, in number of keypresses, which subjects traded for face viewtime.

### Magnetic Resonance Imaging

All functional MR imaging was performed on a Siemens Trio 3 Tesla MRI system using an eight-channel phased-array receive-only RF coil. Subjects were positioned in the MRI scanner and their heads stabilized using foam pads and adjustable paddles fixed to the RF coil assembly. Blood oxygenation level-dependent (BOLD) functional images were acquired using gradient-echo EPI (TR/TE/α 2.5 s/30 ms/90°, 3.125 mm × 3.125 mm × 3 mm resolution), with slices situated parallel to the AC–PC line, and parallel to the inside curve of the FOC to minimize signal distortion in this region (Deichmann et al., 2003). Structural images were acquired using a high resolution T1-weighted MPRAGE sequence (192 sagittal slices over the full head volume, matrix = 224 × 256, FOV = 224 × 256 mm^2^, thickness = 1 mm, no gap) before functional scanning. Details of the imaging parameters and protocol have been reported previously (Perlis et al., 2008; Gasic et al., 2009).

### Data Analysis

#### Behavioral Data

**Figure 3:**
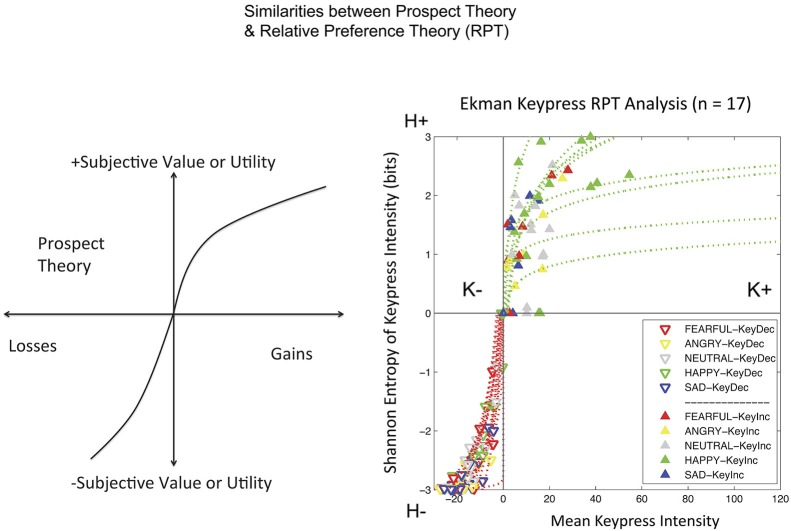
**Relative Preference Graphs**. The output of the keypress paradigm is shown for 17 subjects on the right. The variable K stands for the mean number of keypresses made for category of picture viewed (mean viewtime per category can also be substituted to produce comparable results). K can be interpreted as the effort expended to approach (+ keypresses) or avoid (keypresses) stimuli, and approximates wanting. The variable H stands for the Shannon entropy (information or uncertainty related to making a choice) for each category of picture. It can be interpreted as representing memory about the uncertainty related to making choices over this stimulus set. The approach graph is in green and the avoidance graph is in red. On the left is shown a schema for prospect theory (Kahneman and Tversky, 1979) to emphasize the similarity, despite different variables, between the two theoretical frameworks of prospect theory and relative preference theory (Breiter and Kim, 2008; Kim et al., 2010). Both show an increased slope for negative or avoidance responses relative to positive or approach responses.

**Figure 4:**
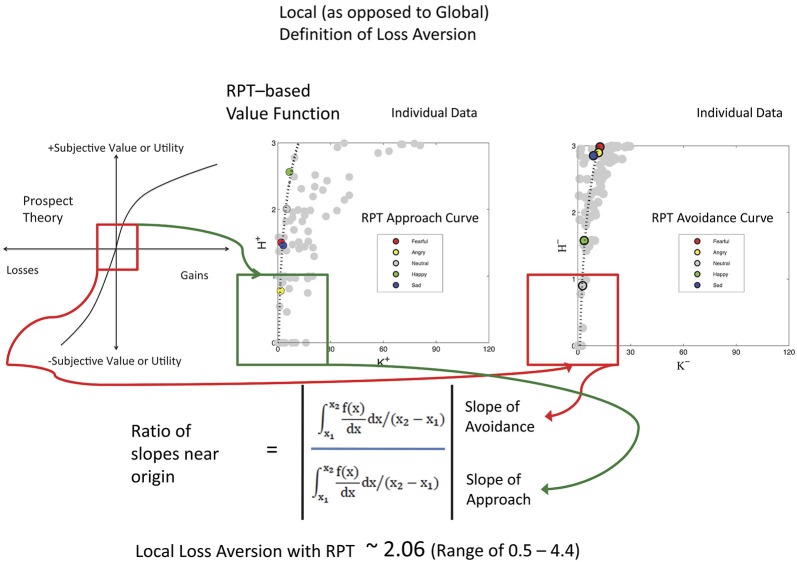
**Local Loss Aversion Computation**. This study used a local rather than a global assessment of loss aversion, meaning that the ratio was computed using the slopes around the inflection point for each graph, rather than the entire graph (Abdellaoui et al., 2007). In this figure, the same cartoon for prospect theory used in Figure 3 is shown on the left, with a red box around the inflection point between the positive and negative graphs that meet at the origin. The approach KH graphs (variables defined in Figure 3 legend) and avoidance KH graphs for the 17 subjects are shown as unfitted points in gray to the right. One example subject is shown with color points as defined in each graph with a fitted curve. The lowest 10% of these curves was then used for assessing the slopes as shown in the mathematics at bottom. Of interest, the LA value for these subjects approximates that reported by Tversky and Kahneman (1992).

Keypress data were checked by a relative preference theory analysis of each subject, using previously validated procedures (Breiter and Kim, 2008; Kim et al., 2010; Figure 3). These procedures produce a valuation graph with variables K and H that encode mean keypress number and Shannon entropy (i.e., information) (Shannon and Weaver, 1949). This valuation graph has been interpreted to relate “wanting” of stimuli (Aharon et al., 2001) to the uncertainty associated with making a choice (Kim et al., 2010). Using a local and general definition of LA (Abdellaoui et al., 2007), we computed the slope of the negative value/utility function (s−) and the slope of the positive value/utility function (s+), to produce s−/s+ (Figure 4). Specifically, s− and s+ were computed by the integral of the curve-fit slope over the 10% of the curve closest to the inflection point or origin (Figure 4). An absolute value of s−/s+ was then computed for each subject. With the full dataset of these subjects we then assessed the association of LA (i.e., |s−/s+|) with age using linear regression; given this test was done in parallel with another test against age (see NDS and age below), there was a correction for multiple comparisons imposed of *p* < 0.05/2 = 0.025.

#### Imaging Data

**Figure 5:**
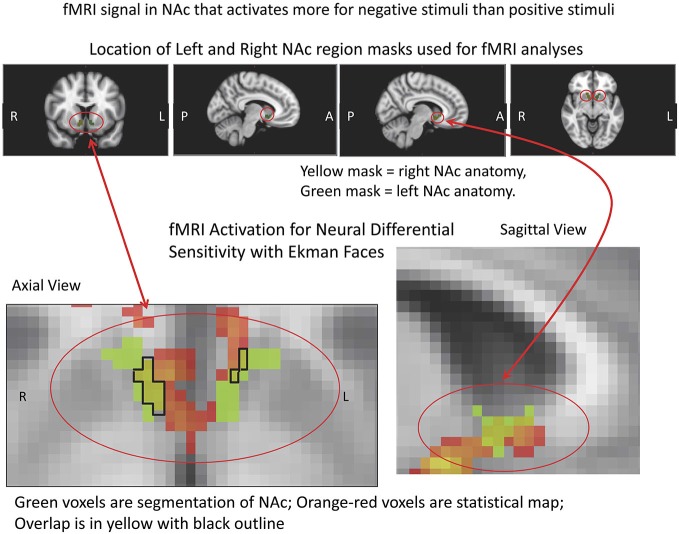
**fMRI Masks and Activation for Neural Differential Sensitivity (NDS)**. The region of interest mask used for the VS/NAc is shown above in color with red circles, with the statistics for the subtraction of negative stimuli vs. positive stimuli in pseudocolor shown below. The segmented VS/NAc is shown in light green in the images below, superimposed on the gray-tone ICBM152 T1 template. The light green segmentations are overlaid with the orange-red statistical map for NDS. Overlap of segmented anatomy and statistical activation map is outlined in black, with yellow inside the closed line.

**Figure 6:**
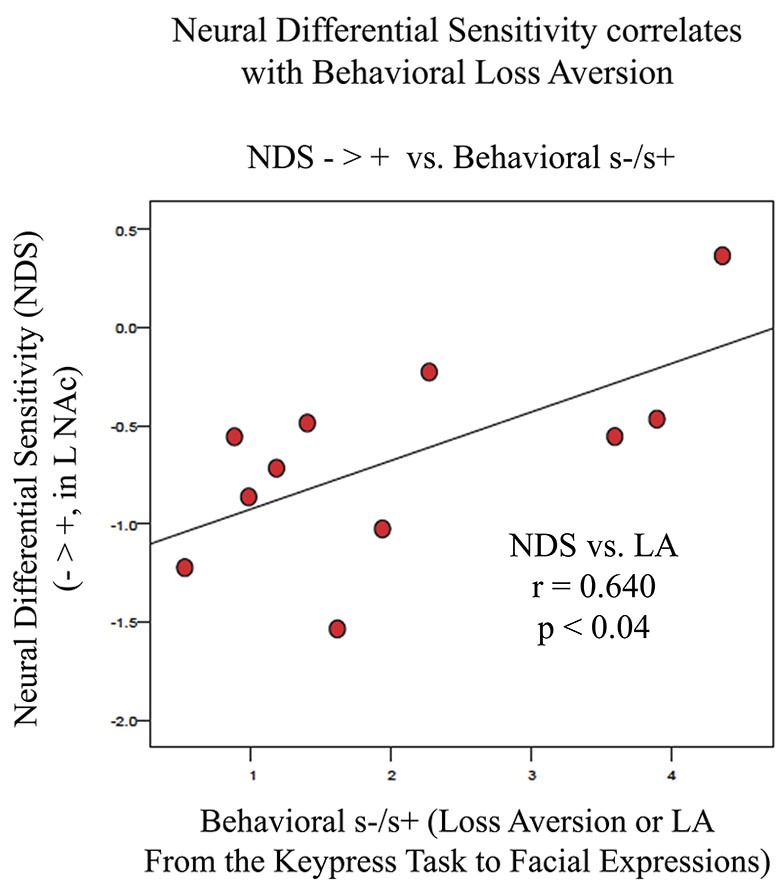
**Association between LA and NDS from BOLD signal collected by fMRI**. NDS represents the difference between BOLD signal in the left VS/NAc for negative stimuli (Angry, Fearful, and Sad faces) minus positive stimuli (Happy faces). LA is represented as the absolute value of s-/s+ from the relative preference graphs of each subject as shown in Figure 3. The line of best fit is shown from this association.

**Figure 7:**
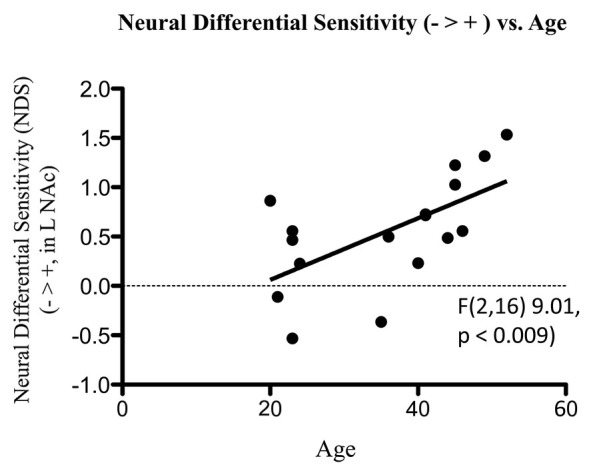
**Association of NDS from BOLD signal collected by fMRI with age**. The line of best fit is shown from this association.

fMRI data were analyzed using the FSL platform (FMRIB’s Software Library, v4.1.9)^2^, and followed signal processing and statistical analysis procedures we have detailed elsewhere (Perlis et al., 2008; Gasic et al., 2009). Stimuli were grouped based on keypress responses into stimuli subjects avoided (Angry, Fearful, and Sad faces) and stimuli subjects approached by using the keypress task to increase viewing time (Happy faces). These two stimuli classes were contrasted in order to determine brain areas that responded more highly to negative (avoidance) than to positive (approach) stimuli [i.e., the β slope for the negative activation (or PE for the –COPE) was greater than the β slope of the positive activation (or PE for the +COPE)]. Statistical maps of NDS to losses relative to gains (− > +) were constructed as a group map, and voxels selected above a whole brain correction for z-stat = 2.3 that overlapped the VS/NAc segmentation volumes from the ICBM152 T1 template (Perlis et al., 2008; Gasic et al., 2009; Figure 5). VS/NAc segmentation followed previously published parameters for its boundaries (Breiter et al., 1997), using processes that have been well validated (see Breiter et al., 1994; Makris et al., 2004). As a second step in our model-based fMRI analysis, we assessed the correlation of NDS [(Angry, Fearful, and Sad > Happy)] in the VS/NAc to LA, as described by Tom et al. (2007), using a subset of the 17 subjects who were not statistical outliers (Figure 6). For this isolated correlation, significant effects had to meet *p* < 0.05. With the full dataset of these subjects we then assessed the regression of NDS with age (Figure 7), and LA with age. Given two assessments against the age variable, significant effects had to meet *p* < 0.05/2 = 0.025.

## Results

### Behavioral Data

All 17 subjects produced keypress data with value function graphs consistent with relative preference theory (Breiter and Kim, 2008; Kim et al., 2010; Figures 3, 4). All graphs produced LA computations (Figure 3), although five subjects had s-/s+ ratios that were > 2 standard deviations above or below the cohort mean, and thus were considered outliers. The LA estimate for remaining subjects was 2.06 + 0.36 (mean + SE) (Figure 4), and the confidence interval of this group overlapped the LA mean of 2.25, published by Tversky and Kahneman (1992). The regression of LA to age showed a non-significant relationship (*p* > 0.1).

### Neuroimaging Data

fMRI data showed significant motion artifacts in two subjects. In the remaining subjects, significant fMRI activation was observed in the VS/NAc bilaterally in the majority of subjects (see segmentation-based masks of the VS/NAc and group statistical map in Figure 5). In individuals, voxels of activation with *p* < 0.05, *z* = 1.96 that overlapped segmentation of the VS/NAc were used to sample BOLD signal representing NDS to losses relative to gains. Across subjects, we found that activation to negative stimuli in left and right NAc was significantly greater than activation to positive stimuli (Figure 5). This signal was used for a control analysis of the correlation of NDS relative to behavioral LA (*r*_(2,11)_ = 0.64, *p* < 0.04). Without outliers (>2 SD from mean NDS), we found the same relationship (Figure 6) reported by others (Tom et al., 2007). When we assessed the relationship of NDS in the VS/NAc to age, we observed a significant positive correlation (*F*_(2,16)_ = 9.01, *p* < 0.009) (Figure 7).

## Discussion

### Synopsis

This study showed that a validated keypress paradigm that allowed subjects to trade effort for view-time of emotional faces (Ekman and Friesen, 1976), produced a relationship between the mean (K, Figure 3) and pattern of keypressing (H, Figure 3) consistent with previous reports using a beauty stimulus set, the International Affective Picture Set, and food stimuli (Breiter and Kim, 2008; Kim et al., 2010). This relationship from the picture-based keypress task produced a ratio between the slope of the avoidance value function (s-) and slope of the approach value function (s+) as a LA measure quite close to that reported by Tversky and Kahneman (1992), who used a monetary decision task.

The keypress-based LA measure correlated with a measure of NDS from the VS/NAc, in similar fashion to that reported by others using a monetary choice task (Tom et al., 2007), and meeting our two criteria for model-based fMRI effects. Although correlation of LA with age was non-significant, the correlation of NDS with age showed a significant positive relationship. Two sides of the three-way correlation test between LA, NDS, and age were significant, suggesting that as individuals age their NDS also increases but their behavioral index of LA does not. The discussion that follows will evaluate potential hypotheses and implications of these findings, along with important caveats.

### Neurocompensation and Other Hypotheses

The relationship between age and NDS in the absence of a significant relationship between age and behavioral LA suggests interesting hypotheses about the neural processing of LA in relation to age. First, Seidman et al. (1998) have shown that subject IQ is inversely related to brain activation levels across individuals, suggesting that the brain compensates with greater neural activity when functional capacity is lower. Given that aging is known to be associated with brain atrophy (e.g., Raz et al., 2005) and cognitive decline (e.g., Kensinger and Corkin, 2008), it is inferred that the functional capacity of the brain declines with age. The findings in this study suggest the hypothesis that as an individual ages, the neural differential between losses and gains increases to achieve the same level of behavioral LA. This perspective is consistent with recent research on aging wherein preserved cognitive function in the context of age-related brain activity may be thought to represent a neurocompensation mechanism (Meunier et al., 2014). Consistent with the neural efficiency (neurocompensation) hypothesis, age-related differences in reward processing have led to evolutionary theories about the changing costs and need to acquire resources over the lifespan. Namely, youth have optimal health and a lack of resources, which may drive the early aggressive pursuit of rewards (Spear, 2000; Somerville and Casey, 2010); while as age progresses, biological decline drives the need to minimize effort and protect what has been acquired (Heckhausen, 1997; Ebner et al., 2006; Heckhausen et al., 2010).

Consistent with these observations, age-related brand loyalty (Lambert-Pandraud and Laurent, 2010) has been attributed to an increased aversion to risk associated with change (Montgomery and Wernerfelt, 1992; Erdem et al., 2006) leading to investigations of age-related changes in decision making and reward processing across the lifespan (Mata and Nunes, 2010; Eppinger et al., 2011, 2012; Mata et al., 2011; Weller et al., 2011; Paulsen et al., 2012a,b; Barkley-Levenson et al., 2013). In the current study, we observed no significant differences in LA behavior, but did find relatively early (i.e., range 20–55 years old) age-related increases in NDS. The parametric increase in NDS with age may indicate additional neural effort is required to obtain the same behavioral outcomes as one ages. Previous studies support this notion; for example, aging populations show bilateral activation patterns within homologous areas of the PFC while their younger counterparts achieve the same performance with singular lateralized activations (Reuter-Lorenz et al., 1999; Cabeza et al., 2002), suggesting compensatory mechanisms in response to neural senescence (Cabeza et al., 2002; Park and Reuter-Lorenz, 2009; Daselaar et al., 2015). In addition, recent imaging work on cognitive function suggests that behavioral outcomes reflect interactions between age, neural efficiency and processing load (Cappell et al., 2010; Vallesi et al., 2011; Turner and Spreng, 2012).

Alternative hypotheses might consider additional age-related asymmetries in the larger decision making network that interact with the VS/NAc in such a way that the response to LA is either uninhibited or amplified. For example, on the other end of the age spectrum, recent imaging work has shown asynchronous developments of the VS/NAc and PFC parallels adolescents’ predisposition to engage in high-risk behaviors (Steinberg, 2008; Van Leijenhorst et al., 2010; Blakemore and Robbins, 2012; Barkley-Levenson and Galván, 2014), however the relationship only becomes apparent when the asynchronous trajectories of PFC and VS/NAc interact within a specific window of development. The observed VS/NAc response to LA may reflect a similar interaction, albeit in areas that did not reach thresholds of activation that changed behavior in the current experiment, either because of the age group examined, sensitivity of the task, or sensitivity of the imaging paradigm. For example, given the limitations of imaging relatively small and deep subcortical structures, particularly in small sample sizes, additional areas may also contribute to age related changes in processing LA, such as functional differences between the dorsal and ventral striatum, where activations in the dorsal striatum may have been sub-threshold in our data, or may only be associated with anticipation paradigms (Tom et al., 2007) related to LA.

### Limitations

The differences in the relationship between age and neural responses vs. age and behavior suggests future work might benefit from examining VS/NAc response against other regions implicated in decision making, which appear to contribute to LA processing (e.g., consider the amygdala findings in Lee et al., 2012, RO1 submission MH098867, and Canessa et al., 2013). In addition, while the sample size for this study is comparable to similar such work (e.g., Tom et al., 2007), much more needs to be done to develop a better understanding of age effects. In this study, we have a sample size of 17 subjects in the age range of 20–55 years. Future studies might include a wider age range to facilitate determining if there are distinct clusters of subjects supporting one or both of the hypotheses regarding age and neural function, and increase the number of participants to obtain a more densely packed gradient of age distributions. With a more comprehensive and dense parametric gradient of age, we speculate that we would see stronger correlations with VS/NAc activation, however we may also find non-linear functions, or a step function based on plateaus within certain groups of ages.

The use of a model-based approach to fMRI in this study also needs to be carefully considered in terms of its pros and cons. Given concerns about VS/NAc involvement with motor preparation (e.g., Florio et al., 1999) and alteration with motor illnesses such as Parkinsonism (e.g., Aarts et al., 2014; Payer et al., 2015), we sought to avoid motoric influence in cognitive imaging studies of the VS/NAc as occurs by definition with monetary choice paradigms (e.g., Tom et al., 2007; Canessa et al., 2013). The implicit assumption in our model-based approach was that emotional responses would drive keypress behavior, and thus the keypress responses would reflect the assessment of the faces being passively viewed in the scanner (see Perlis et al., 2008; Gasic et al., 2009). Such considerations about the use of valuation systems for processing even passively presented stimuli have been discussed by others (e.g., Hayes and Northoff, 2012), and similar considerations have been employed in other model-based fMRI analyses (e.g., Mittner et al., 2014; Wang and Voss, 2014; White et al., 2014; Xu et al., 2015). In this study, the model had two components: (i) testing if NDS would occur in the VS/NAc; and (ii) assessing if NDS-related fMRI signal in the VS/NAc correlated significantly with LA behavior across subjects. It is important to note that the behavioral process studied is one of only two behavioral models (the other being circadian rhythms) that have been tested to Feynman criteria for lawfulness (Kim et al., 2010). Even with such considerations, there is always the possibility that our implicit assumption does not hold, and the keypress behavior outside the scanner relates to a completely different cognitive function than that occurring in the scanner to passive viewing, such as might be involved with the default network.

## Conclusion

In this study we used a more general interpretation of LA, as an overweighting of aversion (toward negative stimuli) relative to approach (toward positive stimuli), and found close concordance with prior published LA measures (Tversky and Kahneman, 1992; Abdellaoui et al., 2007). This LA measure correlated significantly with the differential processing of negative outcomes relative to positive ones by the brain consistent with other studies (Tom et al., 2007; Lee et al., 2012; Canessa et al., 2013). The absence of LA correlation with age, but presence of age correlation with brain differential sensitivity (i.e., NDS) supports a neural efficiency or neurocompensation hypothesis regarding the effects of age on the process of LA. The data from this study may have implications for future research using non-monetary stimuli, such as consumables, marketing options, or market communications. Crossing this consideration with the aging data, the results of our study suggest that marketing communications and brands research that target older adults might focus on the cost of neural processing in marketing communications. Future fMRI work might specifically target an older adult population in order to examine how brain circuits underlying decision-making may be altered in persons over the age of 65. Future fMRI work might also examine the interactions of the nature of information with other variables such as amount of information (Mata and Nunes, 2010) and uncertainty, and thus give us a better understanding of the sub-processes that occur when older adults make decisions.

## Conflict of Interest Statement

The authors declare that the research was conducted in the absence of any commercial or financial relationships that could be construed as a potential conflict of interest.

## Footnotes

^1^http://pgp.mgh.harvard.edu

^2^http://fsl.fmrib.ox.ac.uk/fsl/fslwiki
